# Brain alterations associated with overweight evaluated by body mass index or body fat index in an elderly population: the PROOF study

**DOI:** 10.3389/fendo.2023.1148068

**Published:** 2023-05-26

**Authors:** Radwan Kassir, Pierre Gimet, David Hupin, Claire Boutet, Jean-Claude Barthélémy, Frédéric Roche, Sébastien Celle

**Affiliations:** ^1^ Department of Digestive Surgery, CHU Félix Guyon, Saint Denis, Saint-Étienne, France; ^2^ Physiologie Clinique et de l’Exercice, CHU Saint-Étienne, Saint-Étienne, France; ^3^ Université Jean Monnet, INSERM, U 1059 SAINBIOSE, Mines Saint-Étienne, Saint-Étienne, France; ^4^ TAPE Research Unit, EA 7423, Université Jean Monnet, CHU Saint-Étienne, Service de Radiologie, Saint-Étienne, France

**Keywords:** elderly, obesity, brain, MRI, body mass index

## Abstract

**Background/objectives:**

Obesity is a complex health issue in which the brain plays a role yet to be determined, especially in the elderly. Indeed, in the ageing population, the balance between fat and lean mass is different; thus, the co-influence between the brain and obesity may differ between the elderly and younger subjects. Our main goal is thus to explore the relationship between the brain and obesity using two different approaches to measure obesity: body mass index (BMI) and an index centred on fat mass, the body fat index (BFI).

**Subjects/methods:**

Among the 1,011 subjects of the PROOF population, 273 subjects aged 75 years underwent 3D magnetic resonance imaging as well as dual-energy X-ray absorptiometry to assess fat mass. Voxel-based morphometry was used to explore the local differences in brain volume with obesity.

**Results:**

Higher BMI and BFI were associated with higher grey matter (GM) volume in the left cerebellum. Higher BMI and BFI were mainly associated with higher white matter volume in the left and right cerebellum and near the right medial orbital gyrus. Higher BMI was also associated with higher GM volume in the brainstem, whereas higher BFI was associated with higher GM volume in the left middle temporal gyrus. No decrease in white matter was associated with BMI or BFI.

**Conclusion:**

In the elderly, the relationship between the brain and obesity does not depend on the marker of obesity. Supra-tentorial brain structures seem to be slightly associated with obesity, whereas the cerebellum seems to be one of the key structures related to obesity.

## Introduction

According to World Health Organization, obesity is defined as excessive fat accumulation that may severely impair health. During the last 30 years, the proportion of people with obesity increased not only in adults but also in the elderly. The obesity epidemic has thus become a genuine health problem.

Obesity is a complex health issue that is multifactorial in origin; multiple causes comprise genetic, biological, lifestyle, socio-demographic, and environmental factors (1). Among these factors, the gut–brain axis, a complex system comprising the central, autonomic, and enteric nervous systems, hypothalamic–pituitary–adrenal axis, and gut microbiota, plays a particular role and is involved in the regulation of appetite (2). Apart from these homeostatic factors, eating and appetite are also driven by hedonic factors in which the striatum is highly involved and by cognitive factors, especially by the reward system (3). The brain could thus be seen as an important centre of the onset of obesity.

However, the brain may also suffer because of obesity itself. Indeed, obesity is one of the key factors of low-grade systemic inflammation. Such a chronic inflammatory status can affect brain structures such as the hippocampus, the cerebral cortex, the brainstem, or the amygdala (4, 5).

Bobb et al. and Cole et al. have shown that obesity was associated with an increased incidence of several neurodegenerative disorders such as Alzheimer’s disease as a result of a reduction in the size of the hippocampus, although they were not able to establish whether obesity was the sole cause or the effect of these changes (6, 7). Furthermore, a meta-analysis suggested that the reduction in cognitive performances in obese patients was more specific to executive functions, information processing speed, and memory (8). Other studies have shown a negative relationship between cerebral volume in general or in specific regions (such as the frontal or temporal areas) and overweight (9, 10).

Lately, several meta-analyses (11–13) synthesized the knowledge about obesity and focal brain volume: various areas such as the cerebellum and frontal regions including the orbitofrontal cortex or temporal lobe seem to be associated with obesity. These studies, therefore, point towards a lower cerebral volume in obesity that may be associated with late life with an increased risk of neurodegenerative diseases.

In the three aforementioned meta-analyses (11–13), most of the included papers explored the link between obesity and the brain in young adult patients. It is of note that neurodegenerative diseases and cognitive impairment mostly occur in the elderly; it would be interesting to explore this link in older populations. To our knowledge, only four studies studied elderly patients (14–17) with mean ages between 69 and 75 years. However, the authors used body mass index (BMI) to define obesity and its severity. Body mass index takes into account fat mass as well as lean mass; it is thus a limited indicator of obesity especially in the elderly suffering physiological sarcopenia. Indeed, body composition changes constantly throughout life, with ageing lean mass and muscle mass reduced and fat mass increased (18). Thus, it seems more accurate to use an index based on fat mass measurement rather than on the total mass. For instance, Figley et al. used a bioelectric impedance scale to calculate body fat percentage (19), and Weise et al. assessed fat mass index by dual-energy X-ray absorptiometry (DXA) (20). Although bioelectric impedance and DXA measurements seem to be both associated with increased mortality (21), DXA is today recognized as the gold standard of body composition measurement and fat mass (whole-body) evaluation.

In this study, we thus explored the link between obesity, defined by either body mass index or fat mass index, and brain changes in the elderly.

## Materials and methods

The PROOF study (PROgnostic indicator OF cardiovascular and cerebrovascular events) is a cohort of 1,011 elderly subjects aged 65 years included in 2001 (22). At the inclusion, subjects did not have any past history of vascular or cardiovascular events, and they also were at low risk of cardiac events despite their age.

In 2011, all subjects were contacted for a new full health assessment including cerebral magnetic resonance imaging (MRI), a whole-body DXA measurement, an ambulatory blood pressure measurement, and a biological collection. Volunteers had the choice to undergo or dismiss any of the proposed examinations.

The PROOF study was approved by an Ethics Review Board (CCPRB Rhône-Alpes Loire), and all participants signed a written informed consent for all clinical research procedures. The clinical trial identifier of the PROOF study is NCT00759304.

### DXA and body fat index

Whole-body DXA was recorded using Hologic QDR-2000, software version V5.67A (Hologic Inc., Bedford, MA, USA). Body fat mass and body lean mass have been measured during a single scan using standard procedures.

To overcome the supposed limitations of BMI, we previously developed in our team (23) a new indicator, the body fat index (BFI), which is calculated as the ratio of fat mass (in kg) to height (in meters), squared. Our population was classified using BFI tertiles according to Ntougou et al. (23): BFI < 6.9 kg/m^2^, normal; 6.9 kg/m^2^ ≤ BFI < 9.3 kg/m^2^, moderately increased; BFI ≥ 9.3 kg/m^2^, people with overweight. Classical thresholds for BMI (BMI < 25 kg/m^2^, 25 kg/m^2^ ≤ BMI < 30 kg/m^2^, and BMI > 30 kg/m^2^) were also used.

### Acquisition and treatment using MRIs

Images were recorded at the Saint-Etienne University Hospital on a Siemens 1.5 Tesla MRI instrument. T1-weighted 3D images were used for an anatomical examination. The T1-weighted image parameters had a repetition time of 1,930 ms, an echo time of 3.93 ms, a visual field of 250 × 250, a matrix size of 256 × 256 × 160, and volume sections with a voxel size of 1 × 1 × 1 mm.

All images were visually inspected by an experienced radiologist (CB), and subjects with major anatomical disorders that could invalidate voxel-based morphometry (VBM) analysis, such as meningioma, hygroma, macroadenoma, or subdural haematoma were excluded.

Grey and white matter were analysed using VBM (24) according to statistical parametric mapping (SPM) software. Classical SPM-VBM was performed: segmentation in grey matter (GM), white matter (WM), and cerebrospinal fluid (CSF) compartments, registration using the DARTEL algorithm, modulation, and 8 × 8 × 8 mm^3^ smoothing. Default parameters were used throughout the pipeline. The total intracranial volume (TIV) was therefore calculated from the data collected by summating the GM, WM, and CSF volumes. The mean image was calculated from the resulting images, and thresholded T-maps were overlayed over sections of this mean image.

In the case of GM changes in the cerebellum, a specific SPM toolbox has been developed by Jörn Diedrichsen, which comprises a spatially unbiased atlas template of the cerebellum and brainstem (SUIT) (25). The pipeline includes the isolation of the cerebellum/brainstem complex, the normalization of each isolated complex to the SUIT template, and finally the calculation of voxel-to-voxel statistics.

### Statistics analyses

Classical statistics were produced by Stata Statistical Software: Release 11 (StataCorp LP, College Station, TX, USA). Chi2 test or one-way ANOVA was used to compare population characteristics with BMI or BFI categories.

Regression analyses between grey or white matter partitions and BMI or BFI were produced by the SPM software using multiple regression analysis. Moreover, a one-way ANOVA was used to explore GM or WM changes according to BMI or BFI categories. Sex and TIV were entered as covariates in all VBM analyses. Significant differences in results were defined by either having a p-value inferior to 0.05 at the voxel level after the family-wise error (FWE) correction or having an FWE p-value inferior to 0.05 at the cluster level associated without correction at the voxel level. If necessary, the SUIT pipeline was used with similar parameters.

## Results

A total of 273 subjects had BMI and BFI (DXA-derived measurements) values and valid MRIs. Classical cardiovascular risk factors (fasting blood glucose, triglycerides, HDL and LDL cholesterol, and systolic and diastolic blood pressure) were different in the BMI-defined groups. BFI thresholds could not discriminate the risk factors in our population. However, we noticed a difference in TIV between the three BFI-defined groups (**Table 1**).

**Table 1:** Subjects’ characteristics according to body mass index or body fat index status.

		BMI				BFI			
	Total	BMI 1	BMI 2	BMI 3	p	BFI 1	BFI 2	BFI 3	p
No. of subjects	273	147	105	21		82	97	94	
Sex (M/F)	101/172	47/100	51/54	3/18	0.002	49/33	43/54	9/85	<0.001
Age (years)	75.3 ± 0.9	75.4 ± 0.9	75.2 ± 0.8	75.0 ± 1.1	ns	75.4 ± 1.0	75.3 ± 0.7	75.3 ± 0.9	ns
BMI (kg/m^2^)	24.9 ± 3.7	22.1 ± 2.0	27.2 ± 1.4	32.5 ± 1.9	<0.001	21.8 ± 2.5	24.4 ± 2.7	27.9 ± 3.1	<0.001
BFI (kg/m^2^)	8.6 ± 3.0	7.0 ± 1.8	9.6 ± 2.2	14.9 ± 2.2	<0.001	5.5 ± 0.9	8.0 ± 0.7	11.9 ± 2.2	<0.001
TIV (L)	1.4 ± 0.1	1.4 ± 0.1	1.4 ± 0.1	1.4 ± 0.1	ns (trend)	1.4 ± 0.1	1.4 ± 0.1	1.4 ± 0.1	<0.001
GM volume (L)	0.5 ± 0.1	0.5 ± 0.1	0.6 ± 0.1	0.6 ± 0.0	ns	0.6 ± 0.1	0.5 ± 0.1	0.5 ± 0.1	ns
WM volume (L)	0.4 ± 0.1	0.4 ± 0.1	0.4 ± 0.1	0.4 ± 0.0	ns	0.4 ± 0.1	0.4 ± 0.1	0.4 ± 0.0	ns
Blood glucose (g)	1.0 ± 0.2	0.9 ± 0.1	1.0 ± 0.2	1.0 ± 0.1	<0.001	0.9 ± 0.1	1.0 ± 0.2	1.0 ± 0.1	ns
Triglycerides	1.1 ± 0.5	0.9 ± 0.4	1.2 ± 0.5	1.4 ± 0.6	<0.001	1.0 ± 0.5	1.0 ± 0.5	1.1 ± 0.5	ns (trend)
HDL cholesterol	0.7 ± 0.2	0.8 ± 0.2	0.6 ± 0.2	0.7 ± 0.2	<0.001	0.7 ± 0.2	0.7 ± 0.2	0.7 ± 0.2	ns
LDL cholesterol	1.4 ± 0.4	1.4 ± 0.4	1.3 ± 0.3	1.3 ± 0.4	ns	1.3 ± 0.3	1.4 ± 0.4	1.4 ± 0.4	ns
Mean SBP	117.6 ± 13.0	114.2 ± 11.1	121.1 ± 13.9	125.7 ± 13.6	<0.001	116.5 ± 11.3	116.2 ± 12.9	120.0 ± 14.3	ns
Mean DBP	71.9 ± 7.3	70.8 ± 6.7	73.2 ± 8.0	74.2 ± 6.9	0.02	71.8 ± 7.9	71.3 ± 6.8	72.6 ± 7.3	ns
HT medication (Y/N)	133/123	62/77	57/39	14/7	0.03	37/40	47/44	49/34	ns

Chi2 or one-way ANOVA was used. Only 259 subjects had blood glucose measurements, 258 had HDL/LDL measurements, 256 had HT medication, and 252 had SBP/DBP measurements. SBP and DBP were obtained by ambulatory blood pressure monitoring.

ns, not significant; M, male; F, female; TIV, total intracranial volume; GM, grey matter; WM, white matter; HDL, high-density lipoprotein; LDL, low-density lipoprotein; SBP, systolic blood pressure; DBP, diastolic blood pressure; HT medication, antihypertensive medication.

BMI, body mass index (BM 1, BMI < 25 kg/m^2^; BMI 2, 25 kg/m^2^ ≤ BMI < 30 kg/m^2^; BMI 3, BMI ≥ 30 kg/m^2^).

BFI, body fat index (BFI 1, BFI < 6.9 kg/m^2^; BFI 2, 6.9 kg/m^2^ ≤ BFI < 9.3 kg/m^2^; BFI 3, BFI ≥ 9.3 kg/m^2^).

Both higher BMI and BFI were associated with higher GM volume in the left cerebellum (**Table 2**). Higher BMI was also associated with higher GM volume in the brainstem, whereas higher BFI was associated with a higher volume in the left middle temporal gyrus (**Table 3**).

**Table 2:** Higher grey matter associated with a higher body max index (BMI) or body fat index (BFI).

Side	Brain region	X	Y	Z	Cluster size	Pclust	Pvox
BMI							
**L**	**Cerebellum**	**−15**	**−66**	**−60**	**7**	**0.036**	**0.034**
BFI							
**L**	**Cerebellum**	**−18**	**−76**	**−57**	**15**	**0.029**	**0.019**

Pclust and Pvox are respectively the p-values at the cluster and voxel levels using a family-wise error correction. Results in bold are significant at the voxel level.

R, right; L, left.

**Table 3:** Higher grey matter associated with a higher body max index (BMI) or body fat index (BFI).

Side	Brain region	X	Y	Z	Cluster size	Pclust	Pvox
BMI							
	Brainstem	2	−40	−21	1,137	0.024	0.028
	**Brainstem**	**2**	**−40**	**−21**	**2**	**0.044**	**0.028**
BFI							
**L**	**Middle Temporal Gyrus**	**−57**	**2**	**−24**	**16**	**0.029**	**0.030**

Pclust and Pvox are respectively the p-values at the cluster and voxel levels using a family-wise error correction. Results in bold are significant at the voxel level.

R, right; L, left.

Higher BMI or BFI was also associated with higher WM volume in various areas including the left and right cerebellum and a region near the right medial orbital gyrus (**Table 4**).

**Table 4:** Higher white matter associated with a higher body max index (BMI) or body fat index (BFI).

Side	Brain region	X	Y	Z	Cluster size	Pclust	Pvox
BMI							
L	Cerebellum	−27	−56	−48	589	0.002	0.000
R	Cerebellum	24	−63	−46	784	0.001	0.000
R	Middle frontal cortex	14	44	−21	361	0.005	0.000
	Other zones						
BFI							
L	Cerebellum	−27	−56	−48	498	0.003	0.000
R	Cerebellum	28	−62	−46	756	0.001	0.000
R	Middle frontal cortex	9	38	−20	519	0.002	0.000
Other zones							

Pclust and Pvox are respectively the p-values at the cluster and voxel levels using a family-wise error correction. Results in bold are significant at the voxel level.

R, right; L, left.

We did not observe any WM volume change associated with BMI or BFI.

Using ANOVA, we did not observe any GM difference between BMI and BFI categories.

However, WM differences between BMI and BFI categories occurred in the right and left cerebellum and near the right medial orbital gyrus (**Table 5**).

**Table 5:** White matter differences according to body max index (BMI) or body fat index (BFI) categories.

Side	Brain region	X	Y	Z	Cluster size	Pclust	Pvox
BMI categories							
L	Cerebellum	−30	−54	−44	309	0.004	0.000
R	Cerebellum	24	−63	−46	353	0.003	0.000
R	Medial orbital gyrus (near)	10	39	−21	139	0.011	0.002
BFI categories							
L	Cerebellum	−27	−56	−48	43	0.026	0.002
R	Medial orbital gyrus (near)	14	44	−21	40	0.027	0.007
R	Cerebellum	22	−70	−38	32	0.029	0.026

Pclust and Pvox are respectively the p-values at the cluster and voxel levels using a family-wise error correction. Results in bold are significant at the voxel level.

R, right; L, left.

SUIT analysis allows a more precise exploration of the cerebellum/brainstem. The previously identified GM cerebellar volume decreases associated with higher BMI or BFI were still observed (**Table 6**). As in total brain analysis, a higher GM volume associated with a higher BMI was observed in the brainstem with an extension in the cerebellum. Higher BFI was associated with a higher volume in cerebellar GM, which was not observed using whole-brain analysis (**Table 7**).

**Table 6:** Lower grey matter associated with a higher body max index (BMI) or body fat index (BFI) using the SUIT toolbox.

Side	Brain region	X	Y	Z	Cluster size	Pclust	Pvox
BMI							
R	Cerebellum	22	−60	−57	263	0.001	<0.001
L	Cerebellum	−12	−66	−59	90	0.005	0.002
BFI							
R	Cerebellum	22	−58	−57	159	0.001	<0.001
L	Cerebellum	−18	−84	−53	39	0.009	0.012

Pclust and Pvox are respectively the p-values at the cluster and voxel levels using a family-wise error correction. Results in bold are significant at the voxel level.

R, right; L, left.

**Table 7:** Higher grey matter associated with a higher body max index (BMI) or body fat index (BFI) using the SUIT toolbox.

Side	Brain region	X	Y	Z	Cluster size	Pclust	Pvox
BMI							
	Brainstem/cerebellum	−2	−42	−23	607	<0.001	<0.001
BFI							
R	Cerebellum	4	−54	−25	547	<0.001	<0.001
L	Cerebellum	−14	−64	−33	131	<0.001	<0.001

Pclust and Pvox are respectively the p-values at the cluster and voxel levels using a family-wise error correction. Results in bold are significant at the voxel level.

R, right; L, left.

**Figure 1:**
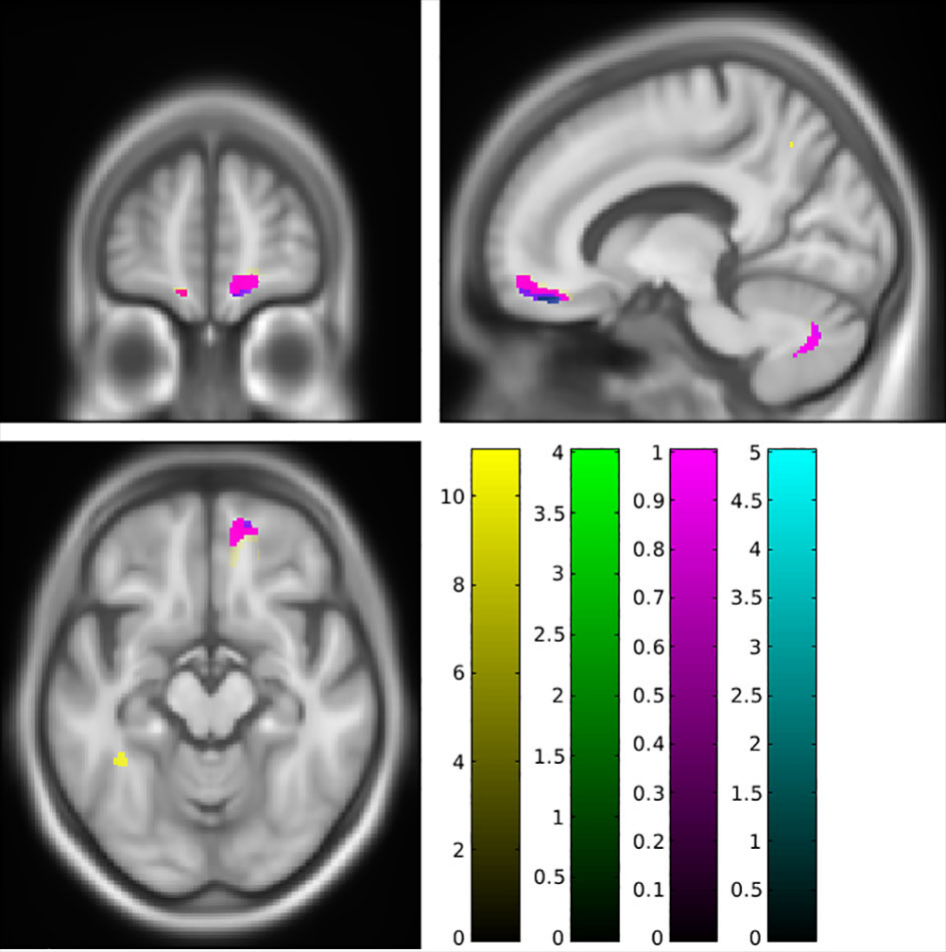
Lower grey matter volume in the posterior cerebellum associated with a higher body mass index (red cluster, pFWE < 0.05) or body fat index (blue cluster, pFWE < 0.05) using SUIT analysis. FWE, family-wise error.

**Figure 2:**
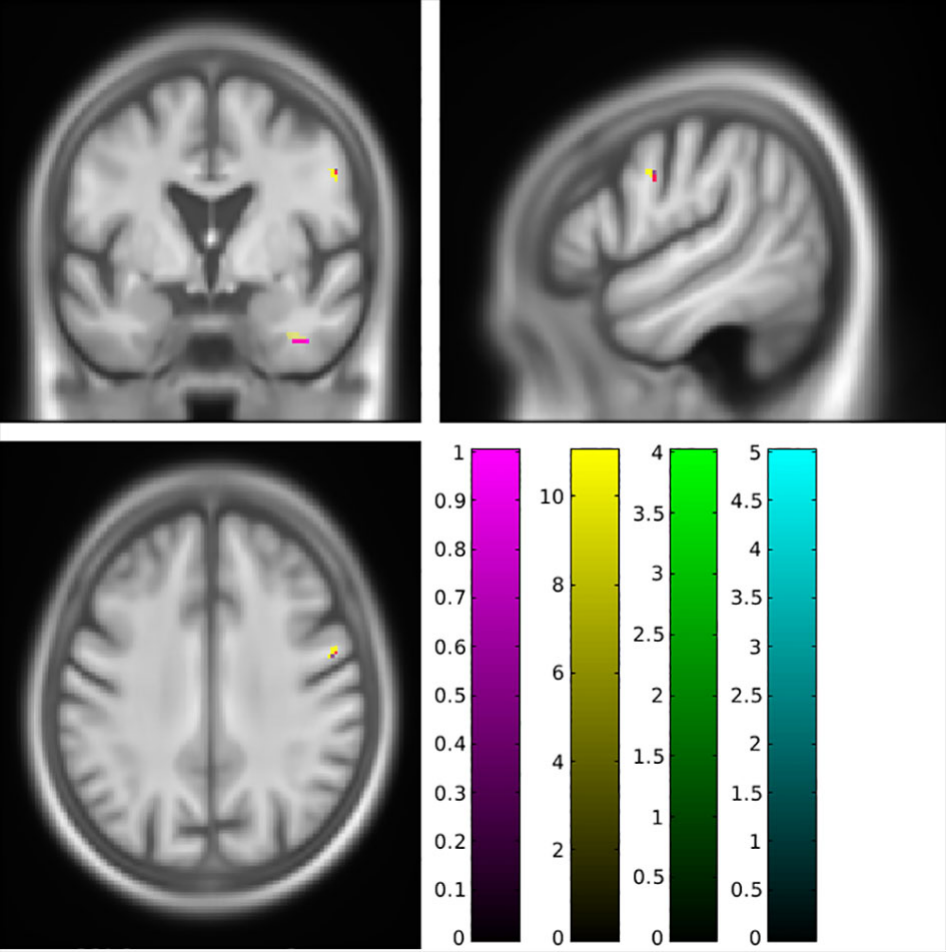
Higher white matter volume in the cerebellum associated with a higher body mass index (magenta cluster, pFWE < 0.05) or body fat index (yellow cluster, pFWE < 0.05) using SUIT analysis. FWE, family-wise error.

**Figure 3:**
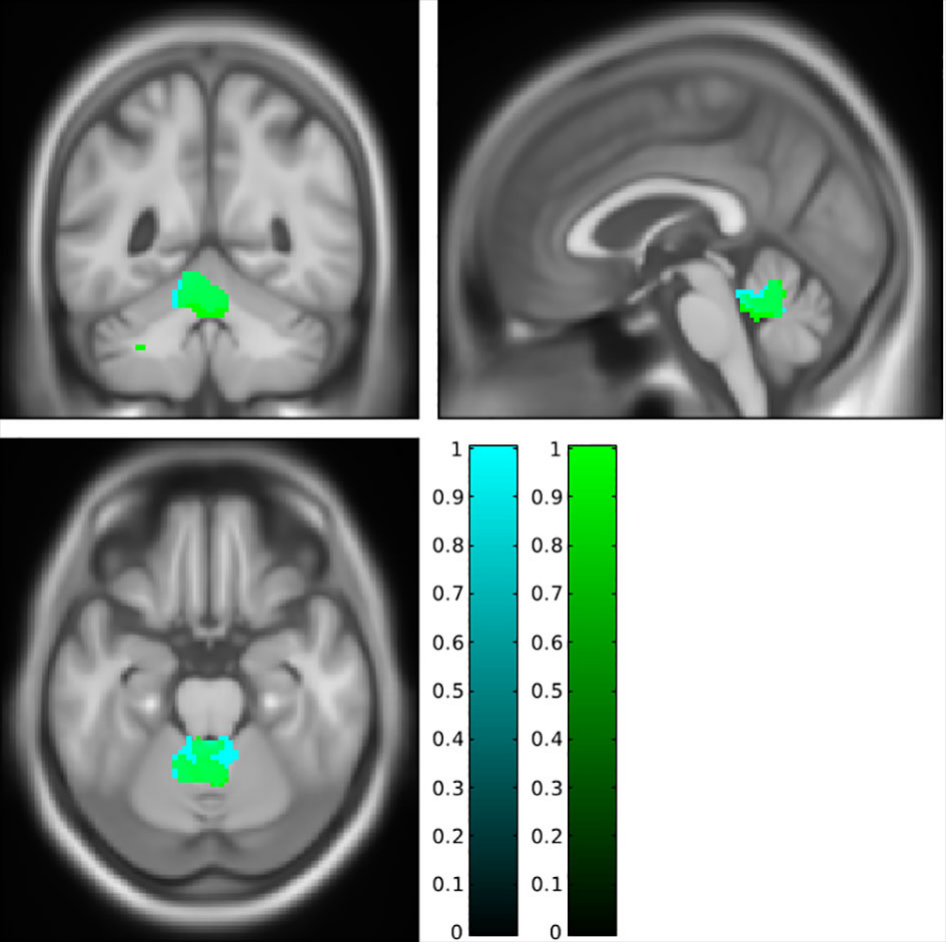
Higher grey matter in the anterior cerebellum with an extension to the brainstem associated with a higher body mass index (cyan cluster, pFWE < 0.05) or body fat index (green cluster, pFWE < 0.05) using SUIT analysis. FWE, family-wise error.

**Figure 4:**
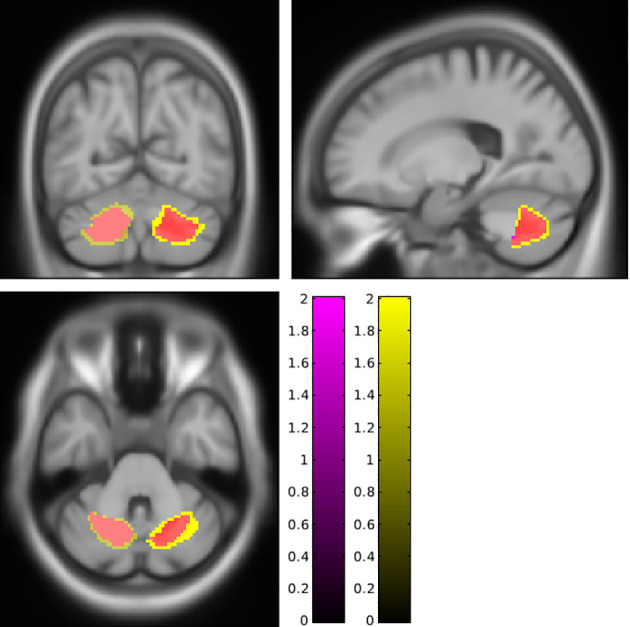
Higher white matter near the right middle frontal cortex associated with a higher body mass index (magenta cluster, pFWE < 0.05) or body fat index (yellow cluster, pFWE < 0.05). This region is also affected when categories of body mass index (cyan cluster, pFWE < 0.05) are explored. FWE, family-wise error.

**Figure 5:**
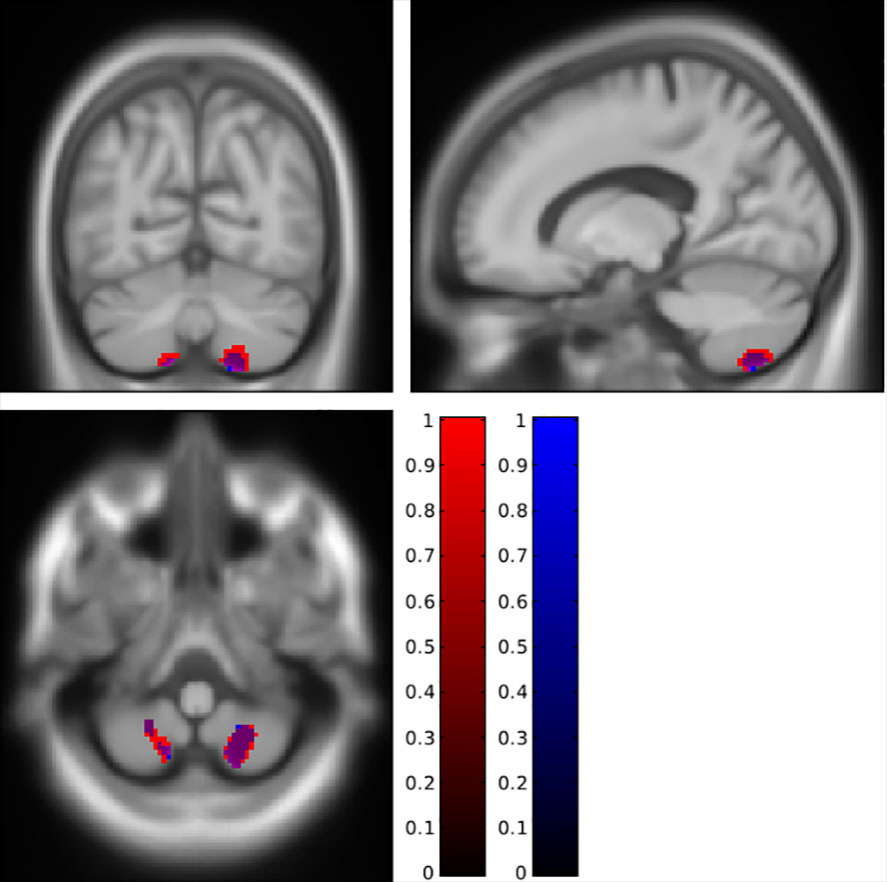
Higher white matter near the right middle orbital gyrus associated with a higher body mass (magenta cluster, pFWE < 0.05) or body fat index (yellow cluster, pFWE < 0.05). This region is also affected when categories of body mass index (cyan cluster, pFWE < 0.05) or body fat index (green cluster, pFWE < 0.05) are explored. FWE, family-wise error.

To synthesize, fat accumulation in our elderly population was associated with five mains areas of grey or white matter changes: two symmetrical regions of higher GM volume in the cerebellum (**Figure 1**; **Supplementary Figure 1**), two symmetrical regions of higher WM in the cerebellum (**Figure 2**; **Supplementary Figure 2**), one region of higher GM volume in the cerebellum with a small extension to the brainstem (**Figure 3**; **Supplementary Figure 3**), a region of higher WM near the right middle frontal gyrus (**Figure 4**), and a region of higher WM near the right middle occipital gyrus (**Figure 5**).

## Discussion

In healthy elderly subjects, body mass index and body fat index were mainly associated with brain differences in the cerebellum and temporal areas. The regions associated with obesity were majorly the same whether we used BMI or BFI as a marker of obesity or adiposity.

In the literature, only two teams were interested in studying anatomic brain defects using another marker of obesity (19, 20). Using DXA, Weise et al. also calculated body fat index but did not explore BMI in their analyses (20). Figley et al. performed a visual comparison between BMI and their marker of obesity, which is body fat percentage (19). In this last study, results obtained by the two techniques partially overlap. Our study is thus the first one to compare the brain defects associated with two separate markers of obesity. Except for the higher GM volume associated with higher BMI/BFI, our results were similar between these two markers of obesity. It thus seems that focusing on fat mass instead of the total mass in the elderly does not change the main conclusion.

The literature linking obesity and GM changes in older people is scarce. To our knowledge, only two studies explored this path (14, 16). Exploring respectively 156 elderly subjects (59 with obesity and 97 without, mean age 75 years) and 42 subjects (8 with obesity, 17 without, and 17 overweight, age range 58–90 years, mean age 69 ± 6 years), Brooks et al. and Schall et al. did not find any lower GM volume between their groups with an appropriate threshold, i.e., with a correction for multiple comparisons (see (26) for some personal advice about the necessity of such a correction). It is noteworthy that Brooks et al. also used a *post-hoc* analysis centred on the frontotemporal regions, thus observing a lower GM volume in the left dorsolateral prefrontal cortex, which is an area involved in appetite regulation. Our study is the first to reveal GM differences associated with obesity in the elderly.

Three meta-analyses (11–13) combining 10, 21, and 25 studies explored the relationship between obesity and brain volume. A reduction of GM related to obesity was observed in various areas including the cerebellum, frontal, and temporal areas. Those meta-analyses did not have any area in common. However, Herrmann et al. and Garcia-Garcia et al. found similar areas in the left cerebellum and the left precentral gyrus (11, 12). Interestingly, we also observed a lower GM volume in the left cerebellum associated with higher BMI or BFI. In a group of older adults (52 to 92 years old), Walther and Masouleh also observed that GM was negatively associated with BMI in the cerebellum (15, 17). In younger subjects, Pannaciulli et al. showed that patients with obesity had a lower GM volume in the cerebellum than lean patients (27). In our study, we also observed a higher WM in the left cerebellum close to the lower GM and a similar higher volume in the right cerebellum. According to Garcia-Garcia, the identified cerebellar areas are connected to prefrontal zones and thus could be involved in cognitive and mood regulation in obesity (12). For Herrmann et al. (11), the cerebellum is also connected to the hypothalamus and is involved in the regulation of eating. Lately, a review by Siciliano et al. (28) focused on the importance of the cerebellum in eating disorders (mainly anorexia nervosa and bulimia nervosa). Cerebellum alteration thus seems to be involved in obesity and its eating regulation

We also observed that GM volume was positively associated with BMI in the middle temporal gyrus. Modifications in temporal areas have been observed previously (11, 12), but they are related to a lower GM volume associated with a higher BMI. Garcia-Garcia et al. advised “caution when interpreting [ … ]” the results from the temporal lobe, but Hermann et al. considered the middle temporal gyrus as a part of the salience network and thus integrating sensory data. This lower GM volume in the salience network in people with obesity as well as an increase in resting state functional connectivity was also observed by Figley et al. (19). However, in our population, observing a higher GM volume, instead of a lower one, is quite unexpected, and no clear explanation can be developed.

We did not observe some of the previously described GM decreases such as in areas in the reward system (12, 29), the inhibitory control system (30), frontotemporal areas (31), or areas dedicated to impulsive/compulsive-related behaviours (29). This may result from differences in subject recruitment (age, mean BMI, and absence of eating disorders) or because of the natural variability of the brain.

Our results are strengthened by the fact that recruitment was based on a cohort population, and this is therefore a large-scale study on the potential relationship between obesity (measured from the BMI and BFI) and cerebral structure (measured by processed MRI images), which excludes any potential selection bias. Furthermore, the population was consistent in terms of age, which excludes any confusion introduced by age-related effects, particularly GM volume. Our study, however, does have limitations: we have few subjects with a BMI over 30 (n = 21, 7.7%), and our results may also be biased by the apparent good health of our subjects. Finally, the limited power of our MRI (1.5 T) may have been insufficient to identify less obvious changes. The major limitation lies in the fact that our study was a horizontal one, and we cannot, therefore, incorporate any time-related parameters into our results.

## Conclusion

Overall, obesity in the elderly, expressed by BMI or BFI, does not clearly correlate with differences in supra-tentorial brain structures, with our main result being a lower volume in the cerebellum, which could be the centre of eating regulation.

Our study is, therefore, encouraging, as we find results that are consistent with those published in the literature, although they are insufficient. Further studies are required to establish better “links” with the limited knowledge on this subject. The ideal study would be a longitudinal one, using multiple covariables, as we have described earlier.

## Data availability statement

The datasets presented in this article are not readily available because a formal request has to be done. Requests to access the datasets should be directed to FR, frederic.roche@univst-etienne.fr.

## Ethics statement

The studies involving human participants were reviewed and approved by CCPRB Rhône-Alpes Loire. The patients/participants provided their written informed consent to participate in this study.

## Author contributions

All authors listed have made a substantial, direct, and intellectual contribution to the work and approved it for publication.

## Supplementary material

Supplementary Figure 1 lower grey matter in the posterior cerebellum associated with a higher body mass index (red cluster, pFWE<0.05) or body fat index (blue cluster, pFWE<0.05).

Supplementary Figure 2 higher white matter in the cerebellum associated with a higher body mass index (magenta cluster, pFWE<0.05) or body fat index (yellow cluster, pFWE<0.05). This region is also affected when categories of body mass index (cyan cluster, pFWE<0.05) or body fat index (green cluster, pFWE<0.05) are explored. Higher volume of white matter in the right inferior temporal gyrus can also be observed.

Supplementary Figure 3 higher grey matter in the brainstem associated with a higher body mass index (cyan cluster, pFWE<0.05).
