# Supplementary figures and images for: Brain alterations associated with overweight evaluated by body mass index or body fat index in an elderly population: the PROOF study
Source: Front Endocrinol (Lausanne). 2023 May 26;14:1148068. doi: 10.3389/fendo.2023.1148068 (PMC10273264; doi:10.3389/fendo.2023.1148068)

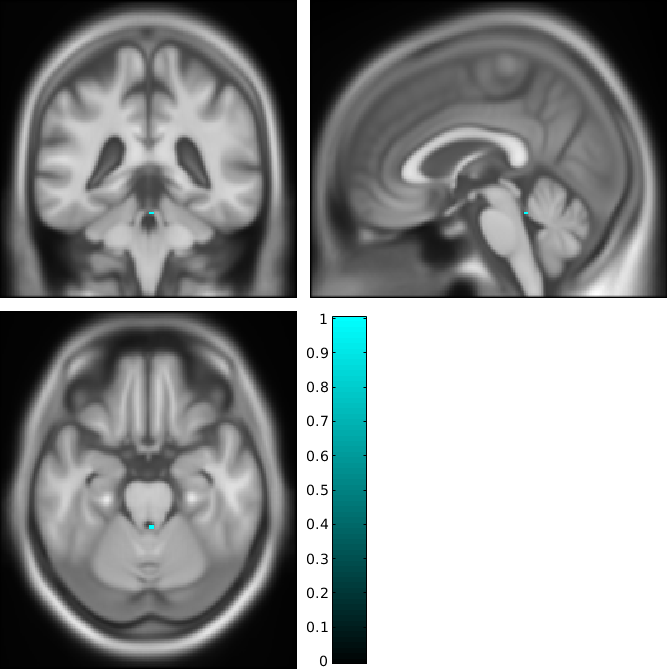

Supplement: Supplementary Figure 1 — lower grey matter in the posterior cerebellum associated with a higher body mass index (red cluster, pFWE<0.05) or body fat index (blue cluster, pFWE<0.05). [file Image_1.tif]

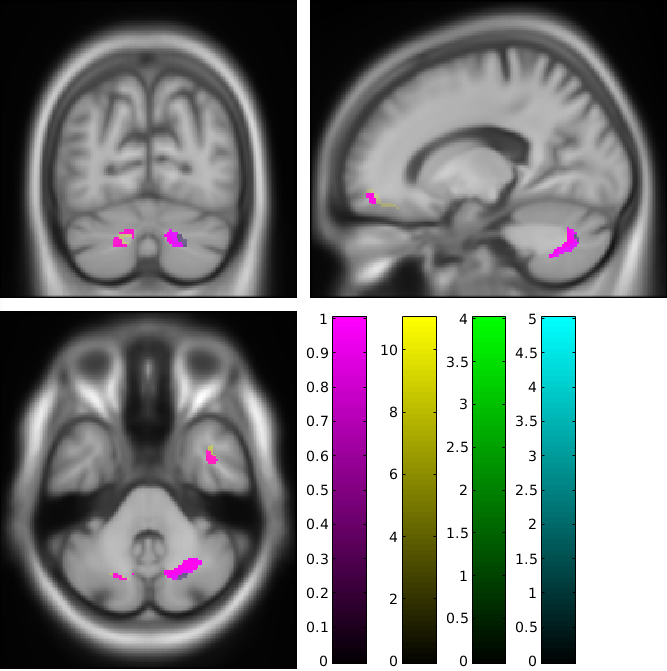

Supplement: Supplementary Figure 2 — higher white matter in the cerebellum associated with a higher body mass index (magenta cluster, pFWE<0.05) or body fat index (yellow cluster, pFWE<0.05). This region is also affected when categories of body mass index (cyan cluster, pFWE<0.05) or body fat index (green cluster, pFWE<0.05) are explored. Higher volume of white matter in the right inferior temporal gyrus can also be observed. [file Image_2.tif]

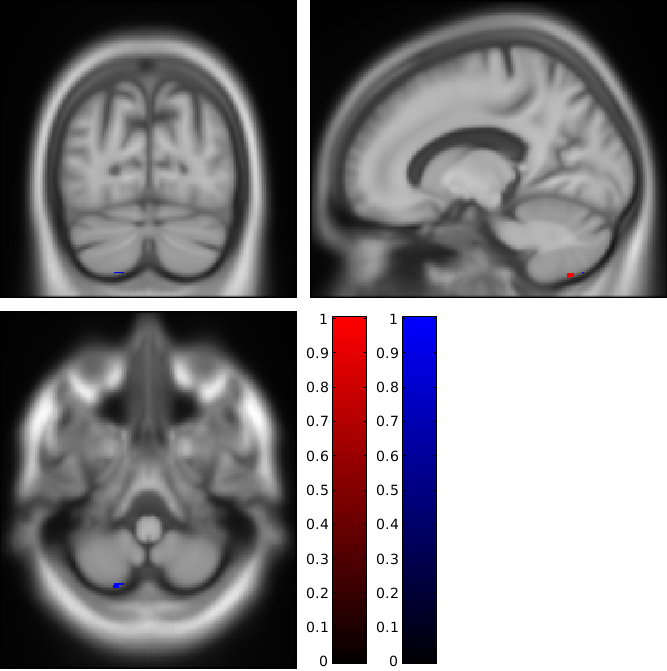

Supplement: Supplementary Figure 3 — higher grey matter in the brainstem associated with a higher body mass index (cyan cluster, pFWE<0.05). [file Image_3.tif]
